# Time series analysis of dengue incidence and its association with meteorological risk factors in Bangladesh

**DOI:** 10.1371/journal.pone.0323238

**Published:** 2025-08-18

**Authors:** Kazi Estieque Alam, Md Jisan Ahmed, Ritu Chalise, Md Abdur Rahman, Tasnia Thanim Mathin, Md Ismile Hossain Bhuiyan, Prajwal Bhandari, Delower Hossain

**Affiliations:** 1 Association of Coding, Technology, and Genomics (ACTG), Sher-e-Bangla Agricultural University (SAU), Dhaka, Bangladesh; 2 Department of Agricultural Economics, Sher-e-Bangla Agricultural University (SAU), Dhaka, Bangladesh; 3 Department of Pathology, Faculty of Animal Science and Veterinary Medicine, Sher-e-Bangla Agricultural University (SAU), Dhaka, Bangladesh; 4 Department of Animal Nutrition, Genetics, and Breeding, Faculty of Animal Science and Veterinary Medicine, Sher-e-Bangla Agricultural University (SAU), Dhaka, Bangladesh; 5 Department of Agricultural Finance and Management, Sher-e-Bangla Agricultural University (SAU), Dhaka, Bangladesh; 6 Department of Medicine and Public Health, Faculty of Animal Science and Veterinary Medicine, Sher-e-Bangla Agricultural University (SAU), Dhaka, Bangladesh; University of Dhaka, BANGLADESH

## Abstract

Dengue is a mosquito-borne viral disease affecting tropical and subtropical regions. In Bangladesh, dengue fever remains a rising public health threat driven by meteorological factors. This study aimed to assess the temporal trends and how meteorological factors influence dengue incidence in Bangladesh from 2008 to 2024. Monthly reported dengue cases were analyzed using time series forecasting techniques and multivariate Poisson regression models. Seasonal Autoregressive Integrated Moving Average (SARIMA) and Extreme Gradient Boosting (XGBoost) models were used for forecasting. Correlation analysis and Poisson regression assessed meteorological effects with one- and two-month lags. The result indicates that the highest number of dengue cases was found in September 2023 (79,598 cases). Autocorrelation revealed a strong positive correlation at 1-month and 2-month lags. Forecasts from 2024–2027 predict that dengue cases will fluctuate between 10,000 and 20,000 annually from the predictive models. Spearman’s rank correlation indicated significant positive associations between dengue cases and precipitation, temperature, wind speed, and humidity. Multivariable Poisson regression revealed that temperature (°C) (IRR = 1.02), Humidity (%) (IRR = 1.25), and Wind speed (m/s) (IRR = 1.10) significantly increased dengue incidence. Between multivariate SARIMA, XGBoost, and Poisson regression, the best-performing model was ARIMA (RMSE: 5058.066). In conclusion, the study highlights the substantial influence of climatic factors on dengue dynamics in Bangladesh, emphasizing the need to integrate meteorological data into early warning systems and develop adaptive, climate-informed control and surveillance strategies.

## Introduction

Dengue is the most significant mosquito-borne viral disease and is found mainly in tropical and subtropical areas. However, due to globalization and climate change, it has spread to countries where it was not common before [1,2]. This disease is caused by four closely related viruses but antigenically distinct virus serotypes (DEN 1–4) of the genus Flavivirus and is transmitted primarily by the *Aedes (A.) aegypti*, the primary urban vector, and *A. albopictus,* the secondary vector [3]. Almost 100 nations in Africa, the Americas, Southeast Asia, the Eastern Mediterranean, and the Western Pacific now have dengue as an endemic disease [4]. More than 50% of the global population is at risk of contracting dengue, with an estimated 50–200 million cases annually [5]. Severe dengue results in approximately 12,500 fatalities each year, primarily in endemic regions where nearly 2.5 billion people are affected [6].

Numerous interconnected elements, including viral serotypes and climatic, hydrological, and societal factors, influence the spread of dengue [7]. Rainfall, temperature, and humidity are examples of meteorological parameters that are closely linked to dengue outbreaks. For instance, it has been demonstrated that higher temperatures and rainfall increase the likelihood of dengue infection by increasing the number of mosquito-breeding sites, improving the mosquito breeding cycle, encouraging vector development (shortening the maturation period), increasing vector survival, and promoting blood-feeding patterns. As a result, there are more vectors in the population, which increases the likelihood that a vector and a human would interact and spread the virus [8,9]. The *Aedes* mosquito has four stages in its life cycle: the eggs, larvae, and pupae are aquatic, and the adult mosquito is terrestrial. Climate variables such as temperature and rainfall affect each stage of the life cycle [10,11]. Research has shown that *A. albopictus* cannot develop at all at a constant temperature of 10°C and that eggs cannot develop at 15°C. However, when the temperature increased to 30°C, the rate at which each stage developed increased steadily, with the fastest development occurring for the larvae and pupae at approximately 30°C [12]. In addition, relatively high temperatures make it possible for mosquito larvae or adults to hibernate [13–16]. The endemic range of Dengue Fever (DF) is expected to spread regionally as long as global temperatures rise [17–19]. Increased reproduction and activity, and a shorter larval incubation period are all made possible by warmer temperatures, which increase the ability to produce progeny. Accordingly, it appears likely that the prevalence and potential for dengue transmission will increase [8,20]. By lengthening the season during which transmission occurs, rising temperatures may also contribute to an increase in dengue transmission [21]. Extended dry spells in endemic regions lacking a reliable source of drinking water could promote water storage, which would increase the number of mosquito breeding grounds used by the *A. aegypti* vector [22].

Consequently, Bangladesh has experienced dengue outbreaks in recent years that have never been severe. Furthermore, Bangladesh is close to nations where dengue is endemic, it is constantly in danger of importing novel strains of the disease [23]. The deadlier secondary dengue infection may have resulted from the introduction of a new serotype through antibody-dependent enhancement (ADE) [24,25]. The first DF was officially identified in 1964 in Bangladesh [26]. The country experienced its first dengue outbreak during the monsoons of 2000, which resulted in 5551 confirmed cases and 93 deaths [23]. A sudden rise in dengue outbreaks was found in 2018 (10,148 cases and 26 deaths), 2019 (101,354 cases and 179 deaths), and 2023 (321,179 cases with 1,705 deaths) [27,28]. Multiple factors contribute to the proliferation of dengue vector habitats in Bangladesh, such as high densities of infected mosquitoes, limited population immunity to circulating dengue serotypes, and poor housing conditions characterized by inadequate waste management, sanitation, drainage, and access to clean water [29–31]. The use of unsafe water storage containers further exacerbates the problem. Additionally, the country’s tropical monsoon climate, marked by heavy rainfall (record highest rainfall in 2023) and favorable temperatures, creates ideal conditions for mosquito breeding, thereby increasing the frequency of dengue outbreaks.

Over the past ten years, a growing amount of research has been conducted to anticipate infectious diseases, specifically dengue and malaria. Traditional techniques, which look for a linear association between underlying causes and disease occurrence, such as regression [30] or autoregressive integrated moving average (ARIMA) [32] Still, achieves a limited level of accuracy. Moreover, contemporary methods such as Support vector machines (SVMs) [33] Artificial neural networks (ANNs) [34,35], and Hidden Markov Models (HMMs) [36] have been developed. The absence of a novel or more robust modeling approach in some studies limits potential advancements in predictive [37–40]. Furthermore, few studies have done predictive modeling like XGBoost for comparing the models and model accuracy, but those studies did not predict the long-term cases, also some of the studies are from other regions, not in Bangladesh [41–45].

Previous studies examining the relationship between climate factors and dengue incidence in Bangladesh have focused on specific regions, particularly Dhaka, and have relied on outdated datasets. This limits their ability to capture recent national trends in dengue transmission. Moreover, most of these studies have not employed predictive modeling techniques, and only a few have utilized basic count regression approaches. The absence of machine learning-based forecasting and comprehensive statistical modeling underscores the need for updated, nationwide research that integrates recent data and robust predictive tools. Such studies are essential to better understand the evolving dynamics of dengue outbreaks under changing climatic conditions across Bangladesh [46–51]. Furthermore, the unusual weather patterns due to climate change, particularly the record rainfall in 2023, have significantly exacerbated the dengue situation in Bangladesh. Thus, this study aims to analyze temporal trends in dengue incidence in Bangladesh and forecast future outbreaks until 2027 using the SARIMA model and XGBoost. Additionally, it examines the influence of dengue-related meteorological factors: temperature, rainfall, humidity, and wind speed, through multivariate Poisson regression. We hypothesize that dengue incidence in Bangladesh is significantly influenced by meteorological factors. The study’s findings will aid public health authorities in developing climate-informed early warning systems and targeted dengue control policies.

## Materials and methods

### Data collection

For this research study, data on monthly dengue cases from January 2008- November 2024 were obtained from the Institute of Epidemiology, Disease Control and Research (IEDCR) website (https://iedcr.portal.gov.bd/site/page/45aea1fa-5756-4feb-8d09-f0da895a3baa/-), Dhaka, Bangladesh [52,53]. The IEDCR provides regularly updated public health data, which ensures the accuracy and relevance of the dengue case statistics used in the study. Similarly, meteorological variable measurement data from January 2008- November 2024 were collected from the Bangladesh Meteorological Department (BMD) (https://bmd.gov.bd/), a reliable platform offering access to a range of environmental and meteorological data of Bangladesh. The data we collected were recorded via the following units: temperature measured in degrees Celsius (°C), rainfall recorded in millimeters (mm), relative humidity expressed as a percentage (%), and wind speed measured in meters per second (m/s). The dataset used in this study was complete, with no missing months or data gaps during the study period. Although the model incorporates key climatic and epidemiological predictors, socio-economic variables such as income, education, and urbanization levels were not included due to data unavailability. This is acknowledged as a limitation, as these factors are known to influence disease dynamics and healthcare access.

### Statistical analysis

#### Descriptive.

A general descriptive summary and box-and-whisker plots were used to analyze the spread time and determine the dengue case distribution in Bangladesh. A box plot provides a visual representation of the distribution. The box extends from the bottom hinge, which is the 25^th^ percentile, to the top hinge, which is the 75^th^ percentile. The line across the box represents the median. To make sure there were no zero values in any month, we added 1 to the total number of cases each month. Since the dengue cases data was not evenly spread out, we used a natural log transformation to make it more suitable for analysis. Later, we changed the log-transformed values back to their original form to make the results easier to understand [54].

#### Decomposition.

The seasonal decomposition process based on loess (STL) is used in a variety of study fields, such as the natural sciences, environmental science, and public health, to analyze time series data. SLT separates the long-term, low-frequency variation in the data into three components: trend (the variation in the data over the same period), seasonal (the variation within the same period), and random or remainder (the remaining variation in the data after extracting the trend and seasonal component). The benefits of SLT include its ease of use, reliable outcomes, and potent data visualization. where $Y_{t}$, $T_{t}$, and R_t_ represents the time series data, trend, seasonal component, and random component, respectively. The following is a description of the equation.


$$Y_{t}=T_{t}+S_{t}+R_{t}$$ (1)

The number of dengue cases in this study is denoted by $Y_{t}$. It is measured on a monthly period. Every year, the number of cases of dengue varies greatly. The numbers during the epidemic year could triple those of the period’s average. As a result, the pattern may be mistranslated. It is imperative to maintain a consistent annual number of dengue cases. As a result, we define a new parameter. $Y_{t}^{*}$, the corrected dengue data are as follows.


$$Y_{t}^{*}=\frac{Y_{t}}{Y_{max}}$$ (2)

where $Y_{max}\;$ is a dengue case of the peak month of the year. We calculated that the dengue endemic would last six months, from January to December. By lessening the impact of outlier cases, the adjusted value enables us to examine the pattern of dengue incidence.

The variance of *Y*_*t*_ can be described as follows:


$$\begin{array}{cc} \text{Var}(Y_{t})= & \text{Var}(T_{t})+\text{Var}(S_{t})+\text{Var}(R_{t})+\text{Cov}(T_{t},S_{t})\\ & +\text{Cov}(T_{t},R_{t})+\text{Cov}(S_{t},R_{t}) \end{array}$$ (3)

The ratio of the variance of the component and the variance of the dataset was calculated as follows:


$$r=\frac{\text{Var}(C_{t})}{\text{Var}(Y_{t})}$$ (4)

#### SARIMA.

An analysis model known as an ARIMA model forecasts potential outcomes via time series data. ARIMA (p, d, q) refers to an ARIMA model that is not seasonal. The number of autoregressive terms, p, is given by the nonnegative integers. The number of times the raw observations are differenced is denoted by d. In the prediction equation, q is the number of lags in the forecast errors. SARIMA (p, d, q)(P, D, Q)m, where m is the number of periods in each season and P, D, Q correspond to the autoregressive, differencing, and moving average terms for the seasonal component, respectively, is an extension of the ARIMA model that incorporates seasonality [55]. In our study, we chose the m value of a 6-month seasonal lag in the SARIMA model, indicating a potential semi-annual autocorrelation structure. This pattern suggests the possibility of sub-annual cycles in dengue incidence, a phenomenon reported in dengue-endemic regions with bimodal climate influences, where dengue peaks have been observed approximately six months apart due to dual rainy seasons [56,57]. Therefore, although SARIMA models with both 6- and 12-month seasonal components were considered, the final model selection prioritizes the 6-month lag to reflect the biologically and climatologically consistent annual dengue transmission cycle, aligning with findings from other tropical regions [58,59].

With the assumption that the causes of these events continue to exhibit the same behavior, these models employ nonseasonal differences, autoregressions, and moving average data from prior samples in addition to seasonal differences, autoregressions, and moving averages from prior periods to accurately predict the subsequent steps of a time series.

A seasonal ARIMA (SARIMA) model is particularly suitable for modeling dengue incidence due to its capacity to capture seasonal components that a conventional ARIMA model may overlook. Based on its structural complexity, the seasonal model can be categorized into two types: a simple additive model and a multiplicative seasonal model. The mathematical form of the simple additive seasonal model is expressed as:


$$X_{t}=S_{t}+T_{t}+I_{t}\;$$ (5)

Where, *S*_*t*_*, T*_*t*_, and *I*_*t*_ denote seasonal information, trend information, and random fluctuation information in the data, respectively.

To construct a SARIMA model for dengue case data, the non-seasonal components are identified first, followed by the seasonal components. A visual inspection of the time series plot of monthly dengue cases is useful to reveal any apparent seasonality pattern. A Box-Cox transformation may be applied to stabilize variance. To remove long-term trends and seasonal effects, first-order differencing and seasonal differencing are employed, respectively. An Augmented Dickey-Fuller (ADF) test is used to check for stationarity in the time series [60,61].

The mathematical formulation of SARIMA models can be generalized as described in Eq. (6).


$$\Delta_{S}^{D}\Delta^{d}Y_{t}=\frac{\Theta_{Q}\left(B^{S}\right)\theta^{q}(B)\epsilon_{t}}{\Phi_{P}\left(B^{S}\right)\phi^{p}(B)}$$ (6)

In this equation, *p*, *d,* and *q* represent the nonseasonal order of autoregression (AR), differentiation, and moving average (MA), respectively. P, D, and Q represent the seasonal order of AR, differentiation, and MA, respectively. Moreover, $Y_{t}\;$ represents the time series data in period *t*. $\varepsilon_{t}$ represents the Gaussian white noise process (random walk) in period *t*. *B* represents the backward shift operator $B^{k}x_{t}=x_{t-k}$. $\Delta_{S}^{D}\;$ represents the seasonal difference, and $\Delta^{d}$ represents the nonseasonal difference. *S* represents the seasonal order.

#### Extreme Gradient Boosting (XGBoost).

Extreme Gradient Boosting (XGBoost) is an optimized and scalable implementation of the gradient boosting framework, designed for high performance in both accuracy and computational efficiency. It is particularly useful for handling complex and large-scale datasets, such as those involved in forecasting dengue cases. XGBoost efficiently evaluates the importance of input features and has demonstrated strong predictive power in various machine learning tasks [62,63]. XGBoost has since undergone significant refinement by the research community [61,64]. The underlying principle of boosting involves combining a series of weak learners (typically decision trees) to form a strong, high-accuracy predictive model. When dealing with complex or high-dimensional datasets, multiple iterations are often required to achieve acceptable predictive accuracy, an area where XGBoost excels [61]. XGBoost uses a regularized objective function that balances model fit and complexity, making it robust against overfitting. The objective function for XGBoost is defined as follows:


$${\text{Obj}}^{\left(t\right)}=\sum {\mathrm{\backslash nolimits}}_{i=1}^{n}l\left(y_{i},{\hat{y}}_{i}^{t-1}+f_{t}\left(x_{i}\right)\right)+\Omega \left(f_{t}\right)+\text{constant}$$ (7)

Where *y*_*i*_ is the observed value, ${\hat{y}}_{i}^{t-1}$ is the predicted value of the last iteration, *x*_*i*_ is the feature vector, n is the sample size, *f*_*t*_ is a new function that the model learns, *Ω*(*f*_*t*_) is the regularization term, which saves the model from complexity. *l* denotes the loss function, which calculates the difference between the label and the prediction in the previous phase, the new tree’s output [60,65].

#### Multivariate Poisson regression (MPR).

Since there is usually a lag of months between changes in meteorological and associated dengue, the goal of this analysis was to determine the best-lagged influence that different meteorological conditions had on dengue occurrence [66]. The interval between a single weather observation and a dengue case was used to define the time lag [67]. The monthly meteorological factors and DF cases were correlated for time lags ranging from zero to two months via Spearman’s rank correlation test. The interval between a single weather observation and a dengue case was used to define the time lag [67].

The following is an example of a basic multivariable Poisson regression model:


$$\ln(Y)=\beta_{0}+\beta_{1}X_{Tmean}+\beta_{2}X_{Precipitation}+\beta_{3}X_{Humidity}+\beta_{4}X_{Wspeed}$$ (8)

The model adjusted for first-order autocorrelation was as follows:


$$ln(Y)=\beta_{0}+\beta_{1}(Y_{t-1})+\beta_{2}X_{Tmean}+\beta_{3}X_{Precipitation}+\beta_{4}X_{Humidity}+\beta_{5}X_{Wspeed}$$ (9)

Here, the variables *X*_*Tmean*_, *X*_*Precipitation*_, *X*_*RHumidity*_, and *X*_*Wspeed*_ represent the mean temperature, amount of rainfall, relative humidity, and wind speed, respectively. All the meteorological variables were tested for multicollinearity with variance inflation factors (VIFs).

Although nonlinear methods can better capture complex relationships in climate-health data, we employed multivariate Poisson regression due to its interpretability and suitability for modeling count data like monthly dengue cases. This approach allowed us to estimate incidence rate ratios (IRRs) for specific meteorological variables, providing actionable epidemiological insights. We acknowledge that Poisson models assume a linear-log link and may not fully capture nonlinear interactions; future studies could integrate machine learning models or generalized additive models (GAMs) to better address these complexities. Thus, the choice of Poisson regression is often a practical and methodologically sound decision when the focus is on understanding variable relationships rather than prediction [50,51].

### Statistical tools and packages

In this study, the R programming language (version 4.4.2) was employed to handle various statistical tasks. Descriptive statistics and regression model summaries were generated via the ‘*gtsummary’* package, which provides easy-to-interpret, publication-ready tables of results. [68]. The ‘*MASS’* package was used to conduct Poisson regression, fitting models for count data [69]. Time series analysis and forecasting were performed via the *forecast* and ‘*dlnm’* packages, enabling decomposition and distributed lag models to understand temporal patterns [70]. For data visualization, the ‘*ggplot2’* package was used to create sophisticated plots [71], while the ‘*corrplot’* package helps in visualizing the correlation matrix effectively, uncovering hidden relationships among variables [72].

### Ethical approval

As this study is based solely on publicly available data, no ethical approval was required.

## Results

### Dengue cases and meteorological factors

There are large variations in the number of dengue cases in Bangladesh, with an average of 3,093 and a standard deviation (SD) of 10,960 from January 2008 to November 2024. Meteorological parameters showed a maximum temperature of 30.47°C (SD ± 3.05°C) and a low of 21.6°C (SD ± 5.2°C), with a wider range of 1.6–27.5°C. The mean temperature showed moderate variability, averaging 25.7°C (SD ± 4.1°C). Precipitation and humidity also vary considerably, while wind speed remains relatively stable (**Table 1**).

**Table 1:** Descriptive statistics of dengue cases and meteorological factors from January 2008 to November 2024 in Bangladesh.

Characteristic	Minimum	Maximum	Average (SD)	Skewness	Kurtosis	median
Dengue Cases (overall period)	0	79,598	3,093 (10,960)	4.96	26.24	80.00
Temperature (°C)	16.0	29.9	25.6 (4.0)	−0.846	−0.75	27.54
Precipitation (mm)	0	817	193 (93)	0.76	−0.46	125.34
Humidity (%)	67.0	89.2	79.7 (5.2)	−0.59	−0.42	80.09
Wind Speed (m/s)	0.57	3.12	1.63 (0.61)	0.46	−0.54	1.56

### Temporal distribution of dengue cases

**Fig 1:**
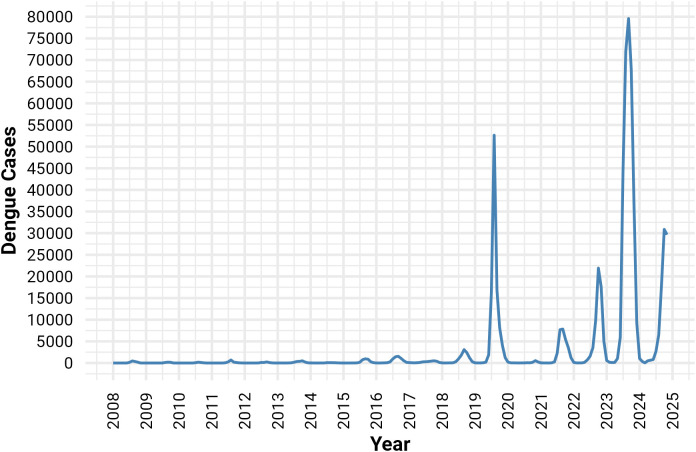
Number of dengue cases from January 2008 to November 2024.

**Fig 2:**
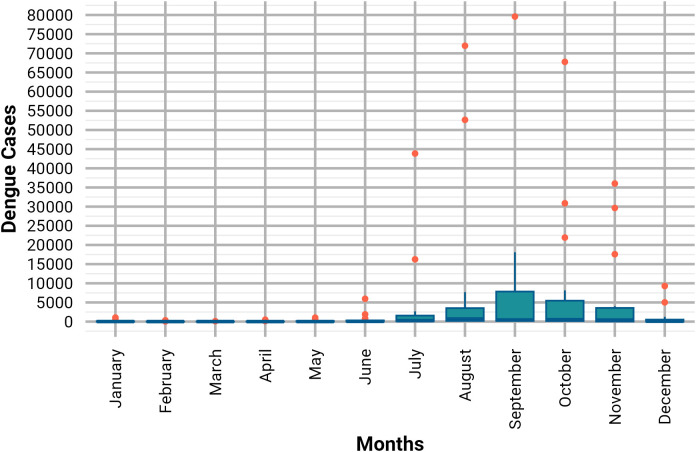
Monthly box plot distribution of the dengue cases.

The highest peak number of dengue cases between January 2008 and November 2024 was found in August 2019 (52,636 cases) and in September 2023 (79,598 cases), along with 71,976 cases in October same year, both years experiencing significant peaks surpassing 40,000 cases (**Fig 1**). The boxplot shows that the maximum number of dengue cases is found from July to November, with September standing out as the peak month for most dengue cases in Bangladesh, and these months also contain outliers of the cases, which were later handled for adjusting the SARIMA model (**Fig 2**).

### Decomposition

**Fig 3:**
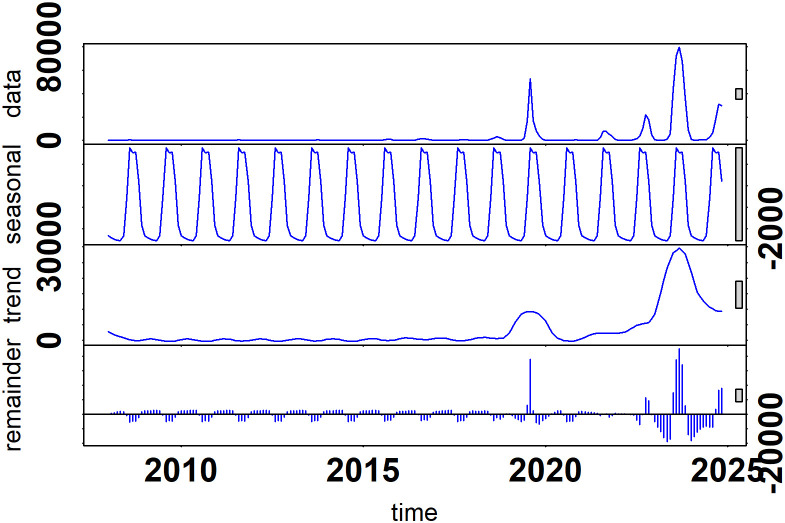
Decomposition plot of the time series of dengue cases in Bangladesh from January 2008 to November 2024.

We created the adjusted data from the raw data by using Eqs. (1) and (2). **Fig 3** shows the STL plot of the raw data. There are prominent high peaks in the data, reflecting significant events, whereas the seasonal component captures strong annual cycles with regular peaks, likely indicating periodic factors. The trend component shows a gradual increase followed by a decline, suggesting greater underlying influences on the data. The remainder of the component displays random fluctuations and spikes, which could represent unexpected events or noise after removing the seasonal and trend effects.

### Autocorrelation and SARIMA

**Fig 4:**
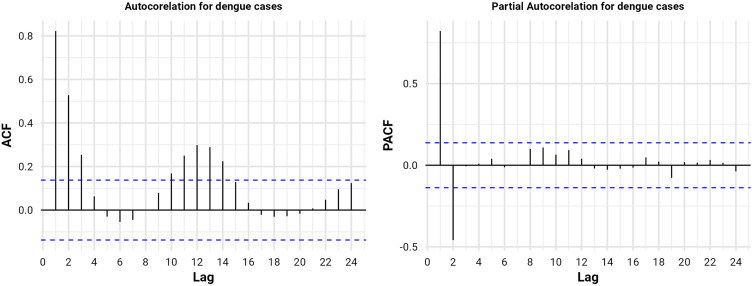
ACF and PACF plots of dengue cases.

The autocorrelation (ACF) and partial autocorrelation (PACF) analyses for dengue cases from January 2008 to November 2024 revealed a strong seasonal pattern (**Fig 4**). The ACF plot shows significant positive autocorrelation at lag 1 (~1 month) and lag 2 (~2 months), with values around 0.8 and 0.5, respectively, indicating that dengue cases are highly correlated with cases from the preceding months. Additionally, moderate autocorrelation is observed around lags 10–12, suggesting possible annual seasonality. The PACF plot exhibits a large spike at lag 1 (~1 month) with a value above 0.5, followed by no significant spikes beyond lag 2, implying that the short-term correlation is primarily driven by the most recent monthly cases. The blue dashed lines in both plots indicate significance thresholds, reinforcing the presence of strong autocorrelations within the first few months.

SARIMA (Seasonal Autoregressive Integrated Moving Average) model identification involves determining the appropriate orders of autoregression (AR), differencing (I), and moving average (MA) terms for both seasonal and non-seasonal components of the time series. Based on the ACF and PACF plots for dengue cases from January 2008 to November 2024, a first-order differencing (*d* = 1) is needed due to the slow decay of the ACF, indicating non-stationarity. The strong spike at lag 1 in the PACF suggests an AR (1) process (*p* = 1 or 2), while the ACF pattern indicates a moving average component (*q* = 1 or 2). The first-order seasonal differencing (*D* = 1). The seasonal ACF and PACF patterns indicate the need for a seasonal autoregressive term (*P* = 1) and possibly a seasonal moving average term (*Q*  = 0 or 1). The 6-month lag was constructed and determined based on autocorrelation (**Fig 4**) and the monthly distribution pattern of dengue cases (**Fig 2**) and based on the cumulative effect of environmental conditions on case occurrence. From the ACF and PACF result 14 SARIMA models can be constructed and the best model was selected by checking the best model selection criteria like mean absolute error (MAE), Root Mean Squared Error (RMSE), and L Jung (p-value) (p > 0.05), and the model selection criteria revealed the following results: Root Mean Squared Error (RMSE): 5203.34, and Mean Absolute Error (MAE) = 1649.56 and determined the best model which is SARIMA (2,1,2) (1,1,1) [6] (**Table 2**).

**Table 2:** SARIMA and XGBoost model characteristics for dengue cases.

SARIMA MODELS	ME	MAE	RMSE	L Jung (*p-*value)
SARIMA (1,1,1) (1,1,1) [6]	81.57	1740.57	5770.859	0.030
SARIMA (1,1,2) (1,1,1) [6]	79.80	1732.75	5762.692	0.044
SARIMA (2,1,1) (1,1,1) [6]	348.48	1622.45	5228.877	0.952
SARIMA (2,1,2) (1,1,1) [6]	349.71	1649.56	5203.34	0.987
SARIMA (2,1,2) (2,1,2) [6]	345.67	1660.55	5203.984	0.971
XG-boost [6]	90.43	190.71	917.49	0.168

**Fig 5:**
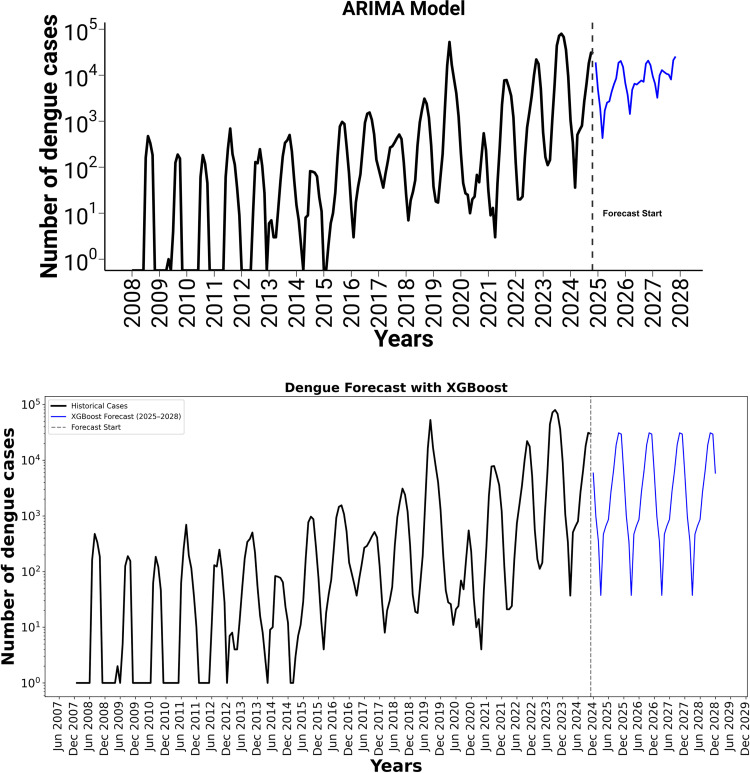
a: Forecast of Dengue Cases (2024–2027) Using Seasonal ARIMA (2,1,2) (1,1,1) [6] Model, b: Forecast of Dengue Cases (2024–2027) Using XGBoost Model.

The prediction from 2024 to 2027 using the SARIMA (2,1,2) (1,1,1) [6] and XG-boost model, where both show periodic oscillations in dengue case numbers every six months (**Fig 5a** and **5b**), indicating a clear seasonal pattern. The forecasted cases range between 10,000 and 20,000, with consistent peaks that suggest recurring outbreaks during specific times of the year. This seasonal regularity highlights the model’s ability to capture the cyclical nature of dengue transmission.

### Correlation analysis of meteorological factors with dengue cases

**Fig 6:**
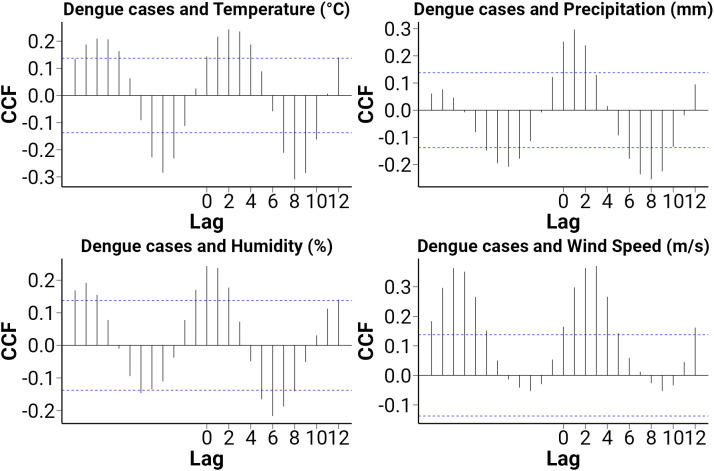
Cross-correlation between Dengue cases and meteorological factors.

**Fig 6** shows the cross-correlation between pre-whitened meteorological variables and dengue cases at lags 0–12 months. Significant positive correlations were observed for temperature at lags 1–5 months, humidity at lags 1–4 months, and wind speed at lags 1–6 months, indicating that these factors may influence dengue incidence after a short delay. As the positive correlations mainly occur between 1–4 months and the dengue transmission period typically spans around 2 months, we selected 1- and 2-month lags for the Spearman correlation analysis.

**Fig 7:**
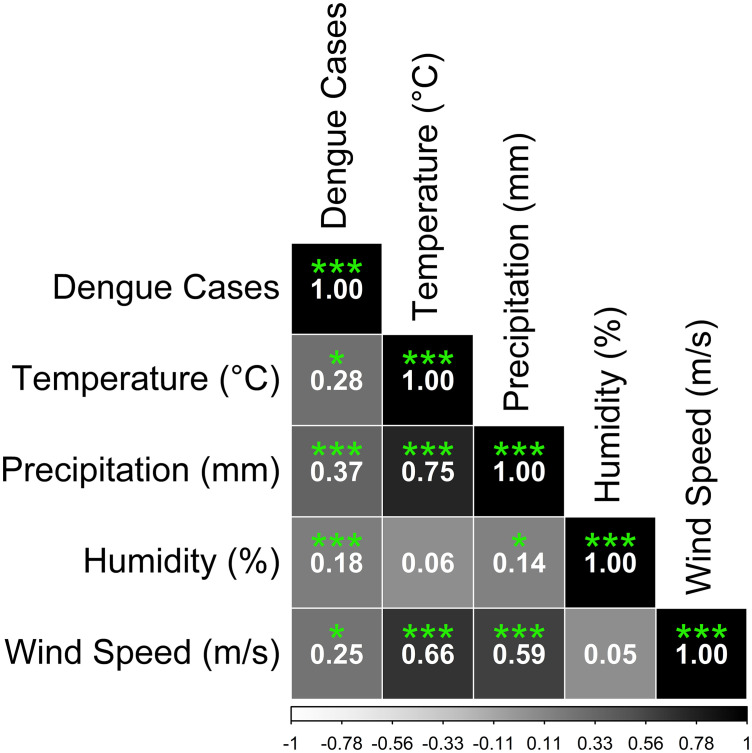
Spearman’s rank correlation plot of the associations of dengue cases with meteorological factors.

Spearman’s rank correlation analysis (**Fig 7**) revealed that dengue cases had the strongest positive correlation with Precipitation (r = 0.37, p < 0.05), indicating that as rainfall increased, so did dengue cases. Temperature (r = 0.28, p < 0.05) and wind speed (r = 0.25, p < 0.05) also exhibited positive correlations with dengue cases, which also indicated the increase in dengue. The relationship between dengue cases and humidity (r = 0.18, p < 0.05) is weaker, but it is significant.

**Fig 8:**
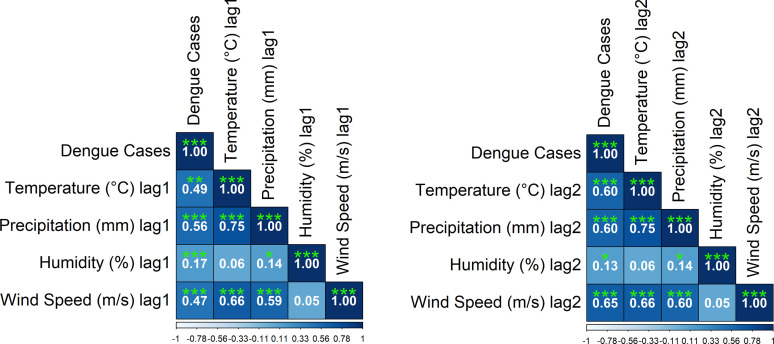
a: Spearman’s rank correlations between meteorological variables and dengue cases in Bangladesh for a one-month lag, b: Spearman’s rank correlations between meteorological variables and dengue cases in Bangladesh with a two-month lag.

The temperature, precipitation, and humidity exhibited significant positive correlations with dengue cases at a one-month lag (**Fig 8**). Specifically, temperature (°C) lag1 showed a moderate positive correlation with dengue cases (r = 0.49, p < 0.001). Precipitation (mm) lag1 displayed a stronger correlation (r = 0.56, p < 0.001), while humidity (%) lag1 had a weaker positive association (r = 0.17, p < 0.001). Wind speed (m/s) lag1 also showed a moderate positive correlation (r = 0.47, p < 0.001). With a two-month lag (**Fig 8**), the associations strengthened. Temperature (°C) lag2 demonstrated a higher correlation with dengue cases (r = 0.60, p < 0.001), and precipitation (mm) lag2 maintained a strong positive relationship (r = 0.60, p < 0.001). Humidity (%) lag2 remained weakly associated (r = 0.13, p < 0.05), while wind speed (m/s) lag2 showed a stronger correlation (r = 0.65, p < 0.001). These findings indicate that temperature and precipitation play a crucial role in influencing dengue outbreaks with a time-lag effect.

### Multivariable Poisson regression

The Poisson regression results indicate that various meteorological factors have significant effects on the cases of dengue (**Table 3**). An increase in temperature (°C) (IRR = 1.02, 95% CI: 1.02–1.02, p < 0.001) is associated with an increase of 2% cases of dengue, likely due to the acceleration of mosquito development and increased virus replication under warmer conditions. Precipitation (mm) (IRR = 1.00, 95% CI: 1.00–1.00, p < 0.001) showed a statistically significant but marginal effect at the per-millimeter level. This suggests that while very small changes in rainfall may have limited immediate impact, larger cumulative increases, such as over 10–100 mm or more, may still influence mosquito breeding and dengue transmission patterns. Additionally, the observed significance may reflect non-linear or threshold effects where moderate rainfall fosters breeding, but excessive rain can reduce larval survival. Humidity (%) (IRR = 1.25, 95% CI: 1.24–1.25, p < 0.001) increases dengue cases by 25%, likely because higher humidity enhances mosquito survival, feeding behavior, and overall vector activity. Wind speed (m/s) (IRR = 1.10, 95% CI: 1.09–1.10, p < 0.001) is associated with an increase of 10% dengue cases, indicating that moderate wind speeds may facilitate mosquito dispersal, potentially increasing the reach of infected vectors.

**Table 3:** Time series Poisson regression of the monthly dengue cases (2008–2024) on the meteorological factors.

Variables	IRR^*1*^	95% CI^*1I*^	p-value
***Temperature (°C)***	1.02	1.02, 1.02	**<0.001**
***Precipitation (mm)***	1.00	1.00, 1.00	**<0.001**
***Humidity (%)***	1.25	1.24, 1.25	**<0.001**
***Wind Speed (m/s)***	1.10	1.09, 1.10	**<0.001**

^*1*^IRR = Incidence Rate Ratio, ^*II*^CI = Confidence Interval; Temperature (°C): Monthly average temperature; Precipitation (mm): Total monthly rainfall; Humidity (%): Average monthly relative humidity; Wind Speed (m/s): Monthly average wind speed.

### Multivariable Poisson regression with lagged

Table 4 presents the Poisson regression results with one-month and two-month lags to assess the influence of meteorological variables on monthly dengue cases in Bangladesh. In the one-month lag model, temperature showed a negative and statistically significant effect on dengue cases (IRR = 0.319, 95% CI: 0.316–0.322, p < 0.001), indicating that higher temperatures in the previous month were associated with lower cases. Precipitation also had a significant negative effect (IRR = 0.002, 95% CI: 0.002–0.002, p < 0.001), suggesting that increased rainfall may reduce dengue transmission, possibly by flushing mosquito breeding sites. Similarly, humidity (IRR = 0.193, 95% CI: 0.192–0.193, p < 0.001) and wind speed (IRR = 0.601, 95% CI: 0.596–0.606, p < 0.001) negatively impacted cases. In the two-month lag model, temperature was still negatively associated with dengue cases but with a weaker effect (IRR = 0.935, 95% CI: 0.930–0.940, p < 0.001). Precipitation (IRR = 0.001, 95% CI: 0.001–0.001, p < 0.001) and humidity (IRR = 0.106, 95% CI: 0.105–0.106, p < 0.001) continued to show significant negative effects. Wind speed also exhibited a negative association with cases (IRR = 0.933, 95% CI: 0.928–0.939, p < 0.001), indicating that stronger winds may disrupt mosquito activity and reduce transmission risk.

**Table 4:** Time series Poisson regression of the monthly dengue cases (2008–2024) with one- and two-month lags on the meteorological factors.

Variables	Poisson regression with one-month Lag			Poisson regression with a two-month Lag		
	IRR^*1*^	95% CI^*1I*^	p-value	IRR^*1*^	95% CI^*1I*^	p-value
***Temperature (°C)***	0.319	0.316, 0.322	**0.000**	0.935	0.930, 0.940	**0.000**
***Precipitation (mm)***	0.002	0.002, 0.002	**0.000**	0.001	0.001, 0.001	**0.000**
***Humidity (%)***	0.193	0.192, 0.193	**0.000**	0.106	0.105, 0.106	**0.000**
***Wind Speed (m/s)***	0.601	0.596, 0.606	**0.000**	0.933	0.928, 0.939	**0.000**

^*1*^IRR = Incidence Rate Ratio, ^*II*^CI = Confidence Interval; Temperature (°C): Monthly average temperature; Precipitation (mm): Total monthly rainfall; Humidity (%): Average monthly relative humidity; Wind Speed (m/s): Monthly average wind speed.

### Model Comparisons with Predictors

Table 5 summarizes the predictive performance of three multivariate models, ARIMA, Poisson Regression, and XGBoost, using climatic variables to model dengue incidence in Bangladesh from 2008 to 2024, where RMSE is considered as the comparison factor. The ARIMA model demonstrated the best predictive performance with the lowest RMSE (5058.066) and was ranked 1st. It identified precipitation and wind speed as key climatic factors. The Poisson Regression model ranked 2nd with a moderate RMSE (8089.84), highlighting temperature, humidity, and wind speed as influential predictors. Conversely, XGBoost performed the poorest (RMSE: 23075.05), ranking 3rd, and identified temperature and wind speed as significant variables.

**Table 5:** Comparative performance of three multivariate predictive models (ARIMA, Poisson Regression, and XGBoost) for forecasting dengue incidence in Bangladesh.

Model	RMSE	Rank	Key climatic factors
Poisson Regression	8089.84	2	Temperature (°C), Humidity (%), Wind Speed (m/s)
ARIMA	5058.066	1	Precipitation (mm), Wind Speed (m/s)
XGBoost	23075.05	3	Temperature (°C), Wind Speed (m/s)

°C = Degree Celsius, m/s = meter/second, % = Percentage

## Discussion

Dengue development, transmission, and incidence are closely tied to climate. As a vector-borne disease, its spread heavily relies on the presence and abundance of its vector. Numerous studies have shown that ecological and climatic factors significantly affect the seasonal occurrence of the dengue virus [55,73–77]. In tropical and subtropical regions, climate change poses a severe threat to global health by enabling the spread of DF. Bangladesh’s total number of dengue cases has risen steadily since 2010, from 101,354 in 2019–321,179 confirmed cases in 2023. Hsan*, et al.* [78] reported that dengue outbreaks in 2019 were intensified by climate change, rapid unplanned urbanization, high population density, and a strained healthcare system. Inadequate preparedness and weak vector control further exacerbate the severity of epidemics. Furthermore, the record-breaking surge in 2023 was driven primarily by climate anomalies and systemic vulnerabilities. Unusually heavy monsoon rains and higher-than-normal temperatures increased mosquito survival and reproduction, while prolonged humidity extended the transmission season [79].

The dengue outbreak in 2023 was unusually severe, with incidence rates more than ten times higher than the average reported during the study period. This spike could lead to significant errors in predictive models, as it deviates considerably from the typical trend. The link between DF and climatic patterns remains poorly understood because of the complexity of the vector and host life cycles. The life cycle of the *A. aegypti* mosquito, the primary vector for dengue, is highly sensitive to climate conditions, which adds to the challenge of fully understanding this relationship [73,80]. However, previous research conducted in South Asia and Southeast Asia has examined the role of climate change in the spread of DF [55,73,81–83]. These studies identified temperature, precipitation, wind speed, and humidity as key climatic factors that may contribute to dengue outbreaks. They also demonstrated the consistent impact of certain climate conditions over time. In this research, we assess the effects of climate variables on dengue incidence, providing valuable insights for mitigating severe future outbreaks and enhancing preparedness efforts in Bangladesh.

The fluctuating trends of dengue fever demonstrate that meteorological factors substantially affect dengue occurrence in southern Thailand [84]. Our research indicates that dengue transmission in Bangladesh transpired from January to December throughout the years 2008–2024. The longest period of dengue transmission in Bangladesh occurred from July to November during the study period. These findings correspond with other studies conducted in southern Thailand [84], which indicated that the most extended dengue transmission season in the Gulf of Thailand occurred from June to September. This aligns with previous findings in Singapore [85], which indicated a rise in dengue incidence from June to October, perhaps because of the geographic and climatic similarities between Thailand and Singapore. Dengue incidence in Delhi, India, peaks in April and from June to October, which is similar to Bangladesh, likely because of the varying monsoon periods between Thailand and India [30]. Moreover, India’s peak temperatures may reach 45°C during the summer months of April, May, and June, hence influencing dengue transmission dynamics [30].

Temperature has been identified as a key predictor for the increase in dengue cases. In this study, a significant correlation was found between dengue incidence from 2008--2024 and factors such as temperature, precipitation, windspeed, and humidity. These findings align with those of a previous study by Pinto *et al*. [82], which showed that minimum and maximum temperatures are strong indicators for predicting dengue case increases on the basis of data from Singapore between 2000 and 2007.

Temperature is the most crucial climatic factor influencing the growth and spread of mosquito vectors, making it a potential predictor of dengue outbreaks [84]. It significantly impacts the entire life cycle and behavior of mosquitoes, including population density, biting rates, gonotrophic cycle durations, and vector size [86,87]. These findings align with earlier research [55,88] confirming that extreme temperatures negatively affect dengue vector development. As a result, temperature influences vector efficiency and the potential for an epidemic [89]. Given the clear link between temperature and dengue incidence, projected temperature changes could intensify dengue transmission in Bangladesh. A possible explanation is that the optimal breeding temperature range for *A. aegypti* mosquitoes is 20–35°C [74]. In our study, the minimum and mean temperatures ranged between 21°C and 30°C, providing ideal conditions for mosquito breeding and facilitating the transmission of dengue viruses.

Another key factor is humidity, which increases the number of dengue patients in Bangladesh. The correlation between dengue incidence and humidity was strongly positive. Similar results were also reported in previous studies [90,91]. This may be attributable to the influence of relative humidity on rainfall, as it indicates the degree of moisture saturation in the atmosphere. A reduction in temperature while the air is saturated can induce condensation, ultimately producing rain [75].

This study revealed that precipitation was associated with dengue cases. In our study, the lag 1 and lag 2 correlations were strongly correlated and were crucial influencing factors in dengue endemics. This outcome is supported by previous studies [92,93]. In Bangladesh, the rainy season lasts from April to September [94]. Rainfall is known to have both positive and negative effects on mosquito growth. On the one hand, light rainfall provides standing water, which is essential for mosquito breeding. On the other hand, excessive or excessive rainfall can disrupt mosquito development by destroying mosquito breeding sites or washing away larvae, limiting their growth potential [95]. In this research, we found that there was a significant increase from July to November, just before the dry season started. Moreover, the peak season for rain is April to June, when the rate is quite low because of extreme rainfall compared with that in later months [73]. This study also revealed that wind speed was weakly positively correlated with dengue incidence in Bangladesh, which is similar to the findings of a previous study [73,96].

In our study, temperature, precipitation, humidity, and wind speed had significant effects on dengue cases between 2008 and 2024. Meteorological factors mostly affect the increase in dengue cases worldwide. The relationships between weather factors and dengue cases may vary across different periods, especially with increasing global warming and urbanization [97]. [98] reported that minimum temperature, humidity, and rainfall were key factors influencing dengue cases, whereas maximum temperature and maximum humidity were not significant in the Mekong Delta area in Vietnam.

In our study, the autocorrelation plot revealed significant positive autocorrelation at the 1-month and 2-month intervals for dengue cases. The virus typically has an incubation period of seven to fourteen days, during which it progresses from an infectious state to an active viral disease. The stability of the dengue virus often remains in endemic equilibrium, influenced by these delay times. Consequently, vector-borne illnesses may persist in carriers for up to one to two weeks [99–101]. The autocorrelation function (ACF) plot was truncated at lag 4, which was parameterized to a moving average (MA) model, with no seasonal lag observed in Nakhon Si Thammarat [102]. In contrast, Islam *et al*. [50] reported that after 12 weeks, the autocorrelation of DF cases gradually decreased to negligible levels. Peaks in the partial autocorrelation were noted at weeks 1 and 4. Chumpu *et al*. [103] also reported that 1-week lag cases had the highest correlation among 65 provinces in Thailand.

In our study, the best-fitting ARIMA model was identified as SARIMA (2,1,2) (1,1,1) [6]. Similarly, Gui *et al*. [97] determined that ARIMA (1,1,0) with first-order differencing and one autoregressive term was the optimal model for capturing the trend and autoregressive properties of the dengue time series. In Bangkok, Polwiang [55] reported that the best-fitting model was SARIMA (1,0,2)(1,1,2) [12], which has a strong seasonal component (1,1,2) combined with a nonseasonal component (1,0,2), indicating a mixed model. For overall dengue incidence in northeastern Thailand, Silawan *et al*. [91] fitted a seasonal ARIMA (2,1,0) (0,1,1) [12] model to data spanning 1996--2003. Additionally, Earnest *et al.* [104] reported that ARIMA (3,1,0) provided the lowest mean absolute percentage error (MAPE) of 19.86, indicating its effectiveness in their study. The validity of our selected SARIMA (2,1,2) (1,1,1) [6] model was assessed through standard model diagnostic checks, including residual analysis and evaluation metrics such as the Akaike Information Criterion (AIC), Bayesian Information Criterion (BIC), and Ljung–Box Q-statistics. These diagnostics confirmed that the model adequately captured the temporal structure of the data without significant autocorrelation in the residuals. However, it is important to note that the model’s predictive validity may be limited by the exclusion of non-climatic covariates such as socio-economic status and urbanization, which are known to influence disease transmission dynamics. Although the SARIMA framework effectively models seasonal and autoregressive patterns, the absence of these explanatory variables may reduce the generalizability of forecasts, particularly at the national level. Future work should aim to incorporate such covariates and explore hybrid models that can better account for multifactorial drivers of disease incidence.

Research has shown that temperature, relative humidity, and rainfall all affect the spread of DF, either directly or indirectly [30,59]. Temperature affects the extrinsic incubation period, reproduction rates, and vector population growth [105,106]. Research has indicated that the confluence of temperature and humidity has a major effect on the quantity of blood consumed and increases the vector survival rate [107]. Numerous studies have demonstrated that the connection between climatic conditions and dengue transmission varies over time and across the Asia-Pacific region [108,109]. The significance of the lag time of climate factors has also been emphasized by numerous studies [110,111]. For example, there was a substantial positive correlation with daily rainfall at a lag of 10 weeks, the minimum temperature at a lag of 1–3 months, the maximum temperature at a lag of 1–4 months, and the relative humidity at a lag of 1–3 months in Taiwan [112].

According to our research, the incidence rates of minimum temperature, relative humidity, and rainfall in Bangladesh were significantly related to one- and two-month lags, respectively. This is also in line with a study conducted in Taiwan [112]. Understanding how climate factors affect *Aedes* mosquito development, maturation, and survival (approximately 7–9 days from egg to adult), as well as how long DENV takes to incubate intrinsically in humans (4–6 days) [113] and extrinsically in the vector (10 days) [114,115] is important. The duration and humidity of dormancy in the environment can impact larval survival, developmental rates, and the production of relatively small adults. Eggs are resistant to desiccation and can endure months of dormancy [116].

In our analysis, we observed a contrasting pattern between non-lagged and lagged Poisson models. While non-lagged models showed positive associations between climatic variables such as temperature and disease incidence (e.g., IRR = 1.02), the lagged models particularly at lag1 and lag2, exhibited IRRs below 1 (e.g., temperature lag1 IRR = 0.319). To investigate whether multicollinearity might explain this inversion, we conducted a Variance Inflation Factor (VIF) analysis. All VIF values were below 1.3 for both lag1 and lag2 models (e.g., Mean_temp_lag1 = 1.259, Precipitation_lag1 = 1.120; Mean_temp_lag2 = 1.201, Precipitation_lag2 = 1.134), indicating no significant multicollinearity. Therefore, this inversion is unlikely due to collinearity or model mis-specification.

Instead, the contradictory IRRs likely reflect true delayed effects of environmental variables. Climatic influences on disease outcomes often operate through complex, time-dependent mechanisms involving vector biology, pathogen incubation, or delayed behavioral responses. For instance, while elevated temperature may enhance immediate transmission risk, it could also lead to subsequent behavioral adaptations (e.g., increased hydration, staying indoors) or changes in vector dynamics that reduce cases after a delay. Similar inverted lag effects have been reported in earlier time series studies. Imai and Hashizume [117] emphasized the importance of considering such temporal dynamics in weather-disease models. Kakarla et al. [118] also showed that climatic variables influence dengue burden differently across lag periods. Likewise, Islam et al. [119] found that childhood diarrhea risks in Bangladesh varied with temperature across different lags, supporting our findings.

One limitation of this study is the exclusion of important socioeconomic and urbanization-related factors that could significantly influence dengue transmission. Variables such as population density, income levels, housing conditions, access to healthcare, and sanitation infrastructure play a crucial role in shaping disease dynamics. Additionally, factors like land use patterns, urban expansion, and climate variations interact with mosquito breeding habitats, further affecting dengue incidence. Incorporating these parameters in future studies could enhance the accuracy and comprehensiveness of dengue risk assessments and predictive models. These covariates could significantly enhance model accuracy and generalizability, particularly for a national-level forecasting framework.

This study has significant ramifications, although it also has considerable shortcomings. The dataset, intended to evaluate the influence of climate variables on dengue incidence in Bangladesh, contains several missing or incomplete records, reflecting inadequate data collection and reporting practices. This may arise from insufficient monitoring mechanisms or a deficiency in awareness among reporting authorities. Consequently, deficiencies in population data may result in biases in estimates due to underreporting or overreporting, given that the dataset encompasses both confirmed and suspected dengue cases.

## Conclusion

This study aims to find the relationship between the seasonal dynamics and dengue in Bangladesh from January 2008 to November 2024. Meteorological variables, temperature, humidity, and wind speed, were found to influence dengue outbreaks. Our findings emphasize the need to integrate real-time meteorological data and consider urbanization and socioeconomic variables in future models. Early warning systems informed by seasonal climate forecasts could guide timely vector control interventions and improve public health response. Strengthening collaboration among meteorological services, health departments, and vector control programs will be essential to operationalize these strategies in the context of a changing climate.

## Supporting information

S1 Table Meteorological data and Dengue cases according to date. (XLSX)

## Abbreviations

DF: dengue fever
SARIMA: seasonal autoregressive integrated moving average
RMSE: root mean squared error
IRR: incidence rate ratio
CI: confidence interval
ADE: antibody-dependent enhancement
ARIMA: autoregressive integrated moving average
SVM: support vector machines
ANN: artificial neural networks
HMM: Hidden Markov models
IEDCR: Institute of Epidemiology, Disease Control and Research
BMD: Bangladesh Meteorological Department
AR: autoregression
MA: moving average
MPR: multivariate Poisson regression
VIF: VARIANCE inflation factors
ACF: autocorrelation function
PACF: partial Autocorrelation function
MAE: mean absolute error
SD: standard deviation
MAPE: mean absolute percentage error
